# Kinship and Leprosy in the Contacts of Leprosy Patients: Cohort at the Souza Araújo Outpatient Clinic, Rio de Janeiro, RJ, 1987–2010

**DOI:** 10.1155/2013/596316

**Published:** 2013-04-10

**Authors:** Daiane Santos dos Santos, Nadia Cristina Duppre, Anna Maria Sales, José Augusto da Costa Nery, Euzenir Nunes Sarno, Mariana Andréa Hacker

**Affiliations:** Leprosy Laboratory, Oswaldo Cruz Foundation, Oswaldo Cruz Institute, Avenida Brasil, 4365, Manguinhos, 21040-360 Rio de Janeiro, RJ, Brazil

## Abstract

A broad variety of factors have been associated with leprosy among contacts, including socioeconomic, epidemiological, and genetic characteristics. Data from 7,174 contacts of leprosy patients from a leprosy outpatient clinic in Rio de Janeiro, Brazil, 1987–2010, were analyzed to investigate the effects of kinship, individual, and contextual factors on leprosy. Multivariate analyses were performed using a robust estimation method. In the prevalence analysis, close kinship (sibling OR = 2.75, offspring OR = 2.00, and other relatives OR = 1.70), socioeconomic factors, and the duration of exposure to the bacillus were associated to leprosy. In the incidence analysis, significant risks were found for all categories of kinship (parents RR = 10.93, spouse, boyfriend/girlfriend, and bride/groom RR = 7.53, sibling RR = 7.03, offspring RR = 5.34, and other relatives RR = 3.71). Once the treatment of the index case was initiated, other factors lost their significance, and the index case bacteriological index and BCG (Bacillus Calmette-Guérin vaccine) protection had a greater impact. Our findings suggested that both genetic susceptibility and physical exposure play an important role in the epidemiology of leprosy, but it was not possible establishing the role of genetic factor. Analyses of other factors related to the genotype of individuals, such as genetic polymorphisms, are needed.

## 1. Introduction

The recorded global leprosy prevalence in 130 countries in the first quarter of 2011 was 192,246 (0.34/10,000 inhabitants), and in 2010, the new case detection was 228,474 (3.93/100,000 inhabitants) [1].

Brazil has the largest number of leprosy cases in the Americas. In 2010, the World Health Organization (WHO) found that out of the 37,740 new cases detected in the region, 34,894 were in Brazil alone where the number of prevalent cases was 29,761 [1].

 The relationship between *M. leprae* and its transmission to the human host and the infection chain leading up to the development of leprosy remains unclear. The long latent period makes understanding the disease transmission difficult. Nonetheless, defining the ways in which these many factors interact with each other may generate a basis for transmission control, which at present partly relies on early diagnosis and treatment [2].

The contacts of leprosy patients are known to have a higher risk of illness than the general population. Contact surveillance is an important strategy to ensure the early diagnosis and control of leprosy. The study of factors associated with leprosy among contacts has identified targets to emphasize for control programs to improve leprosy prevention and control strategies. 

 After infection with *M. leprae, *the development of clinical signs is related to the host's immune profile and certain contextual issues [3]. The factors associated with leprosy constitute a net that includes the molecular biology of the agent, the genetic and immunological characteristics of the host, and social determinants, such as the quality of life, poverty, sanitation, and environmental components [4].

A broad variety of factors have been associated with leprosy among contacts, including socioeconomic and biological individual factors and epidemiological factors related to the index case. Authors have argued that although associating the risk with the degree of intimacy between the patient and contact is common, this association could reflect other mutually shared risk factors, including genetic background [5]. The clinical form [5–8], index case BI [9, 10], consanguinity, and physical proximity [11] have been consistently associated with the risk of disease among contacts.

A Bangladeshi study found a direct correlation between consanguinity with the index case and disease in his/her contact, highlighting the significance of genetic factors, regardless of the degree of physical proximity between these individuals. It has also been argued that interventions geared toward controlling the disease should not only focus on the intrahousehold contacts of the index case but also involve genetically related extrahousehold contacts [11]. 

Previous studies conducted by our group have accessed the epidemiological factors associated with leprosy among contacts, but none have explored the effect of kinship in the development of the disease. The type of household contact, consanguinity with the index case, schooling, bacillary load, clinical form, and BCG (Bacillus Calmette-Guérin) vaccination were found to be related to the chance of developing leprosy among contacts [8, 10]. 

For some decades, several studies in humans have suggested the influence of genetic factors in leprosy, indicating a relationship between the clinical form and kinship. Although many epidemiological studies have explored the relationship between consanguinity and leprosy and comparisons between reports are hampered by methodological differences, few studies have explored the susceptibility of contacts representing various types and degrees of kinship.

 The aim of the present epidemiological study was to assess the relationship of kinship, epidemiological, and social factors, with illness in a cohort of treated leprosy patient contacts under surveillance at a leprosy referral center for the care and research of leprosy by analyzing its database, which covered a 24-year follow-up period.

## 2. Material and Methods

This retrospective study was performed on a cohort of leprosy patient contacts in treatment and/or under surveillance at the Souza Araújo Outpatient Clinic, from the Leprosy Laboratory, Oswaldo Cruz Foundation (FIOCRUZ), Rio de Janeiro, RJ, referral center, which provides ongoing clinical and laboratory care and preventative education on leprosy. The clientele is primarily composed of individuals from the City of Rio de Janeiro, its metropolitan area, and beyond. Most new patients arrive after being referred to our clinic by both public and private health services, but some come spontaneously to evaluate a suspicion of leprosy.

The socioeconomic, clinical, and laboratory parameters generated from the routine procedures performed during the index case and contact follow-up have been recorded in a database since 1987, when tracking officially began. We retrospectively analyzed a total of 7,174 contacts who were evaluated until December 2010.

### 2.1. Definition of Leprosy Patient and Contact

Household contacts were defined as individuals who lived in the same dwelling (i.e., shared the same kitchen or social/recreational area). Nonhousehold contacts were defined as those indicated by the index case as having had other types of associations, such as next-door neighbors, blood relatives, friends, and colleagues. These contacts are scheduled for an initial evaluation at the earliest possible date after the leprosy diagnosis of their index case. 

Contacts diagnosed with leprosy during the surveillance were classified as either prevalent or incident according to the following criteria. Prevalent cases are contacts that are diagnosed with leprosy for the first time upon initial examination, coinciding with the simultaneous diagnosis of their index case. Conversely, incident cases are leprosy contacts (see definition above) who are initially found to be healthy but develop the disease during followup. 

From 1987 to 1991, all leprosy patient contacts were instructed to make an annual visit to the clinic. As of 1992, however, contacts have been advised to return at the first sign of any suspicious-looking skin lesion or nerve impairment. Even so, during treatment or after discharge, an individual may choose to be treated at another health facility. For this reason, a search was conducted to find any cases reported to the National Disease Notification System between 2001 and 2010 via the probabilistic relationship of records using the RecLink program [12]. Three cases of leprosy were found via this database. All contacts who did not return to the clinic were considered healthy. 

### 2.2. Study Variables

Two outcomes were considered in this study: the incidence and prevalence of leprosy among contacts. To evaluate kinship, a variable was constructed that stratified the categories according to consanguinity and degree of kinship: “parent,” “sibling,” “offspring,” other consanguineous relative (“uncle, nephew, grandparent, grandchild, and cousin”), nonconsanguineous relative (“spouse, boyfriend/girlfriend, bride/groom”), and social bond (“friend, coworker, boss, neighbor, stepchild, parent-in-law, brother-in-law, sister-in-law, daughter-in-law, son-in-law, stepmother, stepfather, and godchild”). 

To evaluate other factors associated with leprosy, variables related to the sociodemographic and epidemiological factors tied to individual, index case, and household factors were considered: skin color; childhood BCG, and BCG vaccine (second dose, only in incidence analysis) taken during followup; age; sex; years of schooling; household/nonhousehold relationship; index case variables including bacteriological index (BI) operational classification, and disability grade; and length of time of close association with the index case.

### 2.3. Statistical Analysis

The prevalence and incidence rates were calculated according to the categories of each variable. 

The prevalence analysis excluded incident cases and involved 7,012 contacts. The analysis was performed using logistic regression to obtain the crude and adjusted odds ratio (OR) for the categories of each variable explored.

To evaluate the incidence, the Poisson regression was used to obtain crude and adjusted relative risks (RRs) for the categories of all variables. Coprevalent cases were excluded from the incidence analysis and constituted 6,831 contacts.

 Adjustments were made in the multivariate regression models involving the variables that, in the bivariate analysis, were shown to be statistically associated (at a 5% significance level) with leprosy together with the control variables and epidemiological relevance already established in the literature. The statistical packages SPSS version 16.0 and STATA version 8.0 were used.

Contacts may exhibit characteristics similar to those of their index case due to common exposures to certain environmental conditions; these similarities were taken into account in the statistical analyses by employing robust estimation methods that consider the correlation structure among the contacts within each index case cluster.

## 3. Results

### 3.1. Prevalence Analysis

Incident cases were excluded for the prevalence analysis. This analysis included 7,012 contacts of 1,360 index cases with a mean of 4.8 (SD = 4) contacts per index case. The initial examinations diagnosed 343 (4.9%) contacts as coprevalent.

Results of bivariate analysis are presented in Table 1. The prevalence among contacts by kinship was as follows: parent (8.1%); sibling (8%); offspring (5%); spouse, boyfriend/girlfriend, and bride/groom (4.4%); and uncle, nephew, cousin, grandparent, and grandchild (3.7%). Finally, social contacts and nonconsanguineous relatives presented prevalence of 2.6%. 

A higher prevalence was detected among those aged 15 and older (5.4%), black and/or brown-skinned individuals (5.4%), and those with 4 years of schooling or less (5.9%) (Table 1). 

 The prevalence was higher among household contacts, that is, those sharing the same living quarters as the index case (5.7%), those with close proximity to the index case for a minimum five-year period (5.6%), and those who had not received a BCG vaccine in childhood (8.9%). A higher prevalence was found among contacts of the index case with BI > 3 (7.4%).

 In the multivariate analysis (Table 2), the variable consanguinity was excluded from the final model due to its colinearity with the variable kinship and the operational classification by not showing any significance in the presence of BI. The disability grade was also excluded due to the lack of significance in the bivariate analysis.

 In the final model (Table 2), there was a significant association between prevalence and sibling (adjusted OR = 2.75) and offspring (adjusted OR = 2.00). Moreover, the categories of uncle, nephew, cousin, grandparent, and grandchild continued to have no statistical significance (adjusted OR = 1.70); the categories of parents (adjusted OR = 1.69) and of spouse, boyfriend/girlfriend, and bride/groom (adjusted OR = 1.25) lost significance. 

The presence of a BCG scar continued to have a protective effect against the disease, whereas higher BI, black/brown skin color, schooling of up to 4 years, intrahousehold coexistence, and close proximity to the index case for a minimum five-year period were associated with a higher prevalence.

### 3.2. Incidence Analysis

Coprevalent cases were excluded for the incidence analysis. This analysis included 6831 contacts of 1319 index cases with an average of 5 (SD = 4.1) contacts per index case.

During the study, 162 incident cases were diagnosed. The incidence density for the period was 162/80,406.86 (2.01/1,000 person-year (py)). Regarding kinship, the incidence rates in descending order were the following: parents (4.1/1,000 py); spouse, boyfriend/girlfriend, and bride/groom (2.77/1,000 py); sibling (2.63/1,000 py); offspring (2.05/1,000 py); uncle, nephew, cousin, grandparent, and grandchild (1.49/1,000 py); and social contacts and relatives with no consanguinity (0.47/1,000 py).

A higher incidence rate was observed among women (2.19/1,000 py) and those with black/brown skin color (2.69/1000 py). Higher incidence rates were found among household contacts of the index cases (2.44/1,000 py) and among contacts who did not receive BCG in infancy (2.69/1,000 py) (Table 1).

A higher incidence rate was observed among contacts of the index case with an MB operational classification (2.57/1,000 py) and BI > 3.0 (3.12/1,000 py).

In the multivariate analysis (Table 2), the years of schooling and consanguinity were excluded in the model because they did not exhibit any significance in the bivariate analysis (Table 1). However, a BCG scar was included in the final model due to its epidemiological relevance. 

Regarding the index case variables, the operational classification was not included in the final model because it was not significant in the presence of BI, and the disability grade was omitted due to its lack of association with the outcome. The variables of the duration of the close association with the index case and the household/nonhousehold relationship were excluded due to lack of significance and because they did not show changes with regard to the effects of the other variables in the final model. 

Kinship exhibited a significant association in all categories in the final model with different magnitudes for the relative risk estimations: parents (adjusted RR = 10.93); spouse, boyfriend/girlfriend, and bride/groom (adjusted RR = 7.53); sibling (adjusted RR = 7.03); offspring (adjusted RR = 5.34); and uncle, nephew, cousin, grandparent, and grandchild (adjusted RR = 3.71) (Table 2).

Again, in the final model, black/brown-skinned individuals and BI > 0 maintained a significant association with incidence in the bivariate analysis. The presence of a BCG scar continued to be associated with a protective effect against the disease.

### 3.3. Final Remarks

**Figure 1:**
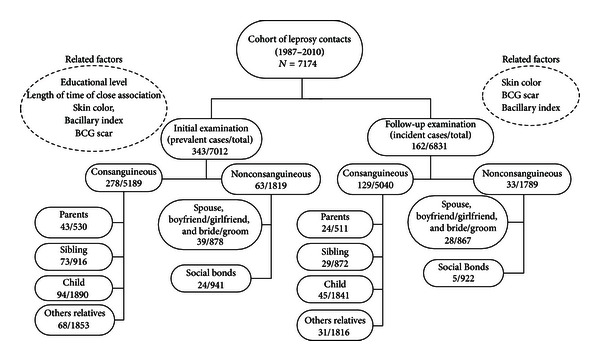
Kinship and leprosy in the contacts of leprosy patients.

As shown in Figure 1, the factors associated with the prevalence include socioeconomic factors and the duration of exposure to the bacillus. With respect to the incidence, once treatment of the index case was initiated, these factors lost their significance, and index case BI and BCG protection had a greater impact on the risk of illness.  In the prevalence analysis, close kinship (offspring and siblings of the index case) showed a significant association. In the incidence analysis, however, a variety of different significant risks were found for all categories of kinship (Figure 1).

## 4. Discussion

The results revealed a significant association among siblings and offspring in the prevalence analysis, indicating that these kinship levels had the highest susceptibility to the disease. Conversely, different magnitudes of association with the disease susceptibility were revealed by the incidence analysis. Associations were found between illness in leprosy patient contacts and nonwhite skin color, exposure to positive index case BI, and the protection effect afforded by the BCG vaccine in childhood. 

Our findings suggested that both genetic susceptibility and physical exposure play an important role in the epidemiology of leprosy. Above all, it must be noted that other factors not considered in this study may also influence illness in contacts. The identification of the most susceptible individuals among leprosy patient contacts by analyzing the factors leading to illness is of extreme importance for the control of leprosy and could serve as a basis for more effective preventive measures against the disease in an effort to strictly control the transmission chain.

Standard techniques involving disease detection and clinical evaluation were assiduously followed and performed by professionals skilled in treating leprosy. As such, the chances of information bias affecting the data were limited. This combination of an ample sample size, abundant reliable data, and an exceptionally lengthy follow-up period provided a unique opportunity to trace, pinpoint, and highlight trends in the epidemic for the purpose of discovering new avenues for research in controlling and preventing the further spread of the disease.

The Clinic Souza Araújo is a reference service, and its cases are especially subject to certain issues, such as difficult diagnosis and suffering some of the most severe forms of the disease. Therefore, this sample does not represent the population of cases and shows an obvious selection bias. Moreover, this sample is constituted by those contacts who are brought by patients, which represents another possible source of selection bias. 

With respect to the relationship between prevalence and kinship, similar results, adjusted for the clinical form, physical distance, and age, were found by Moet et al. [11]. The results of this analysis support the view that a genetic relationship is indeed a relevant risk factor, independent of physical distance. These authors considered the fact that the genetic contribution to the development of leprosy remains to be independent from the effect of relatives living in close proximity. Although, the physical distance was measured merely according to dwelling in this study, close relatives could potentially spend more time together than nonrelated individuals. We agreed with this idea as our prevalence analysis was controlled by physical distance and showed the highest adjusted OR between close genetic relatives, but improving this finding requires the quantification or accurate measurement of the type of contact.

 Durães et al. (2010) found an independent risk of leprosy for two exposures: the type of household relationship and first-degree kinship (father, mother, son/daughter, and sibling) [13]. Although our study was controlled for other variables in the multivariate analysis, there is agreement with the prevalence result that parents, siblings, and offspring have a higher risk of developing leprosy.

In our prevalence analysis, the categories of spouse, boyfriend/girlfriend, bride/groom, and parent lost their significance in the final model. A possible explanatory hypothesis is that it was confounded by the effect of the type of relationship (household/nonhousehold) and the duration of close proximity with the index case, which was controlled and remained significant in the multivariate analysis.

 This result could be confounded by socioeconomic and demographic factors that could not be considered in this study, especially because of the nature of the retrospective design. People living together usually have a similar socioeconomic and education status, live in close proximity, and have a genetic relationship. Establishing the role of each of these factors requires more detailed data.

 However, in the incidence analysis, kinship remained significant in all of the categories in the multivariate analysis, even after controlling for the type and duration of close proximity with the index case (which were not significant in the final model). The relative risk increased in all categories, with the parent bracket demonstrating the highest risk (RR = 10.93).

Household coexistence showed a significant association with prevalence. For incidence, this association was only significant in the bivariate analysis. This scenario, in which the treatment of the index case and other interventions in the cohort had already been performed and the effect of other controlled factors had been included in the final model, may have minimized the magnitude of household coexistence in leprosy transmission among contacts.

Moet et al. (2006) highlighted the increased risk of illness among household contacts [11]. The different methodology, related to the stratification of the physical distance and the cultural and social relations in Bangladesh, which are different from those in Brazil, needs to be taken into account. Duppre (2008) found an OR of 1.59 for household contacts, confirming our prevalence analysis results [14].

The variables “type of close association” and “length of time of close association” did not measure the intensity of the contact because while intrahousehold coexistence may have occurred for a long period of time, the frequency of contact may have been sporadic. When considering the duration of the close association with the index case, there was a significant association with prevalence but none with incidence. Similar results were found in a previous analysis [10] and in another study [15]. 

Skin color in the black and mulatto classifications was associated with prevalence and incidence. Continental populations vary in their susceptibility to disease, most likely due to genetic factors and adaptations to local and selective factors, such as climate, available nutrients, and social factors. In many countries, skin color has traditionally been used in clinical studies and in the identification of pharmacological phenotypes as proxies for geographic ancestry, and Brazil is no exception in this regard [16].

Generally, symptomatic depigmentation is more readily observed as a presenting symptom in darker-skinned persons. Our casuistic is composed mostly of contacts who have a white skin color on the other hand, these patients are examined by experienced specialized professionals using standardized procedures. This characteristic of the reference clinic may minimize this diagnostic bias. 

Our findings corroborate the findings from molecular biology. According to Vanderborght et al. (2007) in the study of the HLA-DR locus, an association of HLA-DRB1 ∗ 15 with a susceptibility to leprosy *per se* was observed in the Brazilian population, with a greater significance in individuals characterized as being African-Brazilian [17]. Moreover, Cardoso et al. (2010) found that the T allele of the IFNG +874 gene protects against leprosy, specifically among those of African descent, which clearly demonstrates the need for further studies on the association between the susceptibility to leprosy and skin color/race [18]. 

The exposure to higher BIs was significantly associated with prevalence and incidence, confirming the relevance of BI in transmitting leprosy among contacts. This finding corroborates those of other epidemiological studies. For example, Jesudasan et al. (1984) found that household contacts of paucibacillary (PB) patients had a lower incidence rate than contacts of multibacillary (MB) patients and that the presence of other coprevalent cases increased the incidence among household contacts [6].

Ranade and Joshi (1995) showed a positive correlation between the index case BI and the attack rate among contacts [15]. Vijayakumaran et al. (1998) showed that the contacts of patients with a BI >2.0 had a relative risk of 3 compared with patient contacts with a BI <2.0 and that the presence of co-prevalent cases in the same household increased the incidence in the cohort from 7.5/1,000 py to 13.4/1,000 py [9]. Another study on the same cohort as that used in the present study found a greater chance of illness among contacts exposed to a BI >3 [10]. Thus, many studies have confirmed that the treatment of bacillary patients is vital for controlling the transmission chain.

In the present study, up to 4 years of schooling was associated with illness in the prevalence but not the incidence analyses. Conversely, Sales et al. (2011) found that education levels were not associated with either incidence or prevalence. The discrepancy in the prevalence analysis may have been influenced by the method applied regarding the inclusion of variables related to the index case in the final model [10]. Different studies have shown a relationship between the number of years in school and leprosy. In addition, ecological studies have shown that low education levels correlate with high incidence rates [19, 20], which have also been observed in a spatial analysis study [4].

Other retrospective studies on the same cohort as that covered in the present study also revealed variations in the findings regarding the protective effect of BCG [8, 21]. However, the methodological differences (sampling, analysis methods, and follow-up time) of these studies must not be ignored. Matos et al. (1999) found a protective effect of 62% among household contacts after adjustments via the Mitsuda test and the clinical form of the index case [8]. Duppre et al. (1998) showed the protective effect of a childhood BCG scar in the contacts of multibacillary (MB) index cases [21].

## 5. Conclusions

The analysis of the contact prevalence, at the moment of the index case diagnosis, enabled the identification of factors associated with leprosy in the absence of the effect of interventions implemented after index case detection, such as the use of polychemotherapy as well as the identification of a profile of the contact with leprosy. The incidence analysis facilitated the identification of other risk factors irrespective of the load of continuous exposure to the leprosy bacillus once the index case had been treated and other control measures, such as the administration of the BCG vaccine to a contact. Based on the data analyzed in this study, the role of kinship in the genetic factors associated with the transmission of leprosy could not be established. However, other factors related to the genotypes of individuals, such as genetic polymorphisms, have been shown to be related to leprosy and need to be further evaluated.

Both genetic susceptibility and physical exposure seem to play an important role in the epidemiology of leprosy. Due to the complexity of the factors involved in leprosy, such as genetic susceptibility, exposure to often asymptomatic, unidentified bacillary individuals, the long latent period, and variability and peculiarities in playing during the incubation period, an ongoing analysis of the behavior of this endemic disease is still required, especially with large prospective cohorts subject to long-term surveillance, as in the present study.

## Figures and Tables

**Table 1:** Frequency distribution and bivariate analysis of the prevalence and incidence cases of a cohort of contacts at the Souza Araújo Outpatient Clinic, Rio de Janeiro, RJ, 1987–2010.

Variables	Coprevalence			Incidence		
	Cases (%)	*N*	Crude OR (95% CI)	Cases (%)	*N*	Crude RR (95% CI)
Sex						
Female	193 (4.7)	4040	1	101 (2.6)	3948	1
Male	150 (5.2)	2972	1.06 (0.85–1.32)	61 (2.0)	2883	0.81 (0.60–1.10)
Age						
0 to 14 years	88 (3.8)	2289	0.70 (0.53–0.93)	56 (2.5)	2257	1.04 (0.75–1.44)
≥15 years	255 (5.4)	4723	1	106 (2.3)	4574	1
Skin Color						
White	148 (3.8)	3926	1	77 (2.2)	3855	1
Brown/Black	155 (5.4)	2861	1.46 (1.14–1.88)	83 (3.0)	2789	1.70 (1.19–2.42)
Educational level						
>10 years	35 (2.5)	1378	1	23 (2.0)	1366	1
4 to 10 years	42 (3.5)	1186	1.41 (0.90–2.21)	28 (2.0)	1172	1.21 (0.66–2.21)
<4 years	263 (5.9)	4443	2.41 (1.65–3.53)	111 (3.0)	4291	1.27 (0.77–2.07)
Kinship						
Social bonds	24 (2.6)	941	1	5 (0.5)	922	1
Spouse, boy/girlfr, br/gr	39 (4.4)	878	1.78 (1.10–2.88)	28 (3.2)	867	5.9 (2.29–15.44)
Parents	43 (8.1)	530	3.37 (2.04–5.57)	24 (4.7)	511	8.79 (3.29–23.47)
Sibling	73 (8.0)	916	3.31 (2.07–5.30)	29 (3.3)	872	5.64 (2.21–14.38)
Child	94 (5.0)	1890	2.00 (1.26–3.17)	45 (2.0)	1841	4.40 (1.17–11.14)
Other consanguineous relatives	68 (3.7)	1853	1.46 (0.89–2.37)	31 (2.0)	1816	3.20 (1.26–8.12)
Type of association						
Nonhousehold	116 (3.9)	3003	1	49 (2.0)	2936	1
Household	227 (5.7)	4009	1.49 (1.17–1.90)	113 (3.0)	3895	1.70 (1.19–2.42)
Time of association						
0–5 years	46 (2.7)	1686	1	33 (2.0)	1673	1
>5 years	297 (5.6)	5326	2.11 (1.51–2.93)	129 (3.0)	5158	1.24 (0.84–1.84)
BCG scar						
No	212 (8.9)	2385	1	79 (4.0)	2252	1
Yes	131 (2.8)	4627	0.30 (0.24–0.38)	83 (2.0)	4579	0.60 (0.44–0.83)
BCG vaccine						
No				82 (3.0)	2532	1
Yes				79 (2.0)	4269	0.83 (0.58–1.19)
Index case form						
Paucibacillary	43 (2.1)	2085	1	14 (1.0)	2056	1
Multibacillary	300 (6.1)	4883	3.11 (2.16–4.46)	148 (3.0)	4731	4.18 (2.40–7.27)
Index case BI						
BI = 0	47 (2.1)	2232	1	16 (1.0)	2201	1
0 < BI < 3	93 (3.4)	2001	2.27 (1.51–3.39)	56 (3.0)	1964	3.28 (1.77–6.05)
BI > 3	203 (7.4)	2733	3.73 (2.59–5.37)	90 (3.0)	2620	4.85 (2.75–8.57)
Index case DG						
0	170 (4.6)	3732	1	81 (2.0)	3643	1
1	98 (5.1)	1915	1.13 (0.80–1.60)	50 (3.0)	1867	1.17 (0.76–1.80)
2	74 (5.5)	1332	1.23 (0.86–1.76)	31 (2.0)	1289	1.02 (0.64–1.62)

Boy/girlfr: boyfriend/girlfriend, br/gr: bride/groom, BI: bacillary index, DG: disability grade.

**Table 2:** Factors associated with prevalence and incidence in a cohort of contacts. Souza Araújo Outpatient Clinic, Rio de Janeiro, RJ, 1987–2010.

Variables	Coprevalence	Incidence
	Adjusted OR (95% CI)	Adjusted RR (95% CI)
Skin color		
White	1	1
Brown/black	1.32 (1.02–1.70)	1.66 (1.14–2.42)
Educational level		
>10 years	1	—
4 to 10 years	1.33 (0.81–2.18)	
<4 years	2.18 (1.42–3.35)	
Kinship		
Social bonds	1	1
Spouse, boy/girlfr, br/gr	1.25 (0.74–2.11)	7.53 (2.51–22.57)
Parents	1.69 (0.97–2.96)	10.93 (3.48–34.27)
Sibling	2.75 (1.65–4.57)	7.03 (2.41–20.46)
Child	2.00 (1.18–3.39)	5.34 (1.74–16.38)
Other consanguineous relatives	1.70 (0.98–2.94)	3.71 (1.24–11.06)
Type of close association		
Nonhousehold	1	—
Household	1.33 (1.00–1.77)	
Length of time of close association		
0–5 years	1	—
>5 years	1.48 (1.02–2.15)	
BCG scar		
No	1	1
Yes	0.30 (0.22–0.41)	0.63 (0.44–0.90)
Index case BI		
BI = 0	1	1
0 < BI < 3	2.54 (1.62–3.98)	3.68 (1.99–6.82)
BI > 3	4.21 (2.78–6.36)	5.27 (2.96–9.38)

Boy/girlfr: boyfriend/girlfriend, br/gr: bride/groom, BI: bacillary index.
